# Sex differences in default mode and dorsal attention network engagement

**DOI:** 10.1371/journal.pone.0199049

**Published:** 2018-06-14

**Authors:** Kelly M. Dumais, Sergey Chernyak, Lisa D. Nickerson, Amy C. Janes

**Affiliations:** 1 McLean Imaging Center, McLean Hospital, Belmont, Massachusetts, United States of America; 2 Department of Psychiatry, Harvard Medical School, Boston, Massachusetts, United States of America; Banner Alzheimer’s Institute, UNITED STATES

## Abstract

Focusing on sex differences is necessary to fully understand basic neurobiological processes such as the engagement of large-scale brain networks involved in attention. Prior work suggests that women show enhanced attention during tasks of reward/punishment relative to men. Yet, sex differences in the engagement of neural networks sub serving internal and external focus has been unexplored in regard to reward and punishment. Using data from a large sample (n = 190) of healthy participants from the Human Connectome Project, we investigated sex differences in default mode network (DMN), dorsal attention network (DAN), and frontal parietal network (FPN) activation during exposure to reward and punishment. To determine if sex differences are specific to valenced stimuli, we analyzed network activation during working memory. Results indicate that, relative to men, women have increased suppression of the DMN and greater activation of the DAN during exposure to reward and punishment. Given the relative roles of these networks in internal (DMN) and external (DAN) attention, this pattern of activation suggests that women have enhanced external attention to reward and punishment. In contrast, there were no sex differences in network activation during working memory, indicating that this sex difference is specific to the processing of reward and punishment. These findings suggest a neurobiological explanation for prior work showing women have greater sensitivity to reward/punishment and are more prone to psychiatric disorders characterized by enhanced attention to such stimuli. Furthermore, given the large sample from the Human Connectome Project, the current findings provide general implications for the study of sex as a biological variable in investigation of reward processes.

## Introduction

Brain responses to both reward and punishment have been studied extensively, providing critical insight into normal and pathological brain states [1–4]. However, there has been limited investigation into how biological factors such as sex influence these processes. Such sex differences are likely, as women generally show more behavioral sensitivity to reward and punishment relative to men [5–7]. Neuroimaging investigation of sex differences in reward and punishment processing have largely focused on limbic brain regions typically associated with reward and emotion [8–9]. However, when considering sex differences in reward/punishment, there is evidence suggesting that sex differences exist outside traditional reward function. Specifically, event related potential studies suggest women engage more attentional resources when performing reward/punishment tasks [7, 10]. Such findings suggest that there may be sex differences in the engagement of large scale attentional neural networks during reward and punishment processing.

Attentional processes are modulated by the suppression and engagement of large-scale neural networks, such as the default mode network (DMN), dorsal attention network (DAN), and frontoparietal control network (FPN) [11–13]. Specifically, the DMN, comprised mainly of the medial prefrontal cortex (mPFC), posterior cingulate cortex (PCC), inferior parietal lobe, and medial temporal lobe, modulates internally oriented attention, such as self-referential processing, recalling one’s past, and planning one’s future [14,15]. On the other hand, the DAN, comprised primarily of the dorsal lateral prefrontal cortex (dlPFC), frontal eye fields, middle temporal motion complex, and superior parietal lobe, modulates externally oriented attention, such as top-down attentional control [11]. Therefore, the DMN is typically suppressed and DAN activated during cognitive tasks requiring external attention, including those involving rewarding stimuli [16–18]. The FPN modulates attentional processes by flexibly coupling with the DMN and/or DAN to support internal and external attention, respectively [13,19]. Prior work suggests that these networks may be differentially engaged in men and women during exposure to valenced stimuli. For example, core DMN regions, such as the mPFC and PCC, show greater activity in men versus women during exposure to monetary and appetitive drug stimuli [20–22], which could be due to higher activation in men or increased suppression in women. In contrast, a core DAN region, the dlPFC, shows greater activity in women versus men during exposure to negative stimuli [23]. It is plausible that these prior regional findings represent sex differences in DMN and DAN engagement during exposure to reward/punishment.

To determine whether there are sex differences in network engagement during the processing of reward/punishment, we used functional magnetic resonance imaging (fMRI) data from a large group of healthy participants from the Human Connectome Project (HCP) [24]. We analyzed DMN, DAN, and FPN activity during the incentive processing task, which involves exposure to monetary reward and punishment. Because women generally show greater attention to valenced stimuli [7,10,25,26], we hypothesize that women will show greater suppression of brain networks related to internal attentional processes (i.e., greater DMN suppression) and greater activation of brain networks related to external attentional processes (i.e., greater DAN activation) during reward and punishment exposure. To determine if sex differences are specific to the processing of valenced stimuli, we also analyzed network activity during an object n-back working memory task (0-back and 2-back) which is known to reliably suppress the DMN and activate the DAN [18,27]. Men and women typically show equivalent behavioral performance on similar n-back tasks [28–31], though similar working memory performance could still be modulated by different brain regions in men versus women [32]. Indeed, a meta-analysis of sex differences during working memory have found that women show greater activation of limbic and prefrontal structures, such as the amygdala, left superior and right inferior frontal gyri, while men show greater activation of parietal areas such as the superior parietal lobule and left precuneus [33]. However, sex differences in attentional networks during working memory have not been investigated. Together, these results will clarify the neurobiological factors underlying sex-specific processing of reward and punishment, and whether these sex-specific processes are specific to reward/punishment or extend to other cognitive domains.

## Methods

### Participants

Secondary analysis of data collected from the first 500 participants in the Human Connectome Project (S500 data release) was done for the present study. Recruitment details for the HCP are provided in Van Essen et al. [24]. Study was approved by Washington University in the St. Louis’ Human Research Protection Office (IRB #201204036, "Mapping the Human Connectome: Structure, Function, and Heritability"). Briefly, individuals were excluded by the HCP if they reported having a significant history of psychiatric disorders, neurological or cardiovascular disease, if they were pregnant, or if they had unsafe metal in their body. Inclusion/exclusion criteria were assessed by a screening questionnaire developed explicitly for the HCP and using the Semi-Structured Assessment for the Genetics of Alcoholism (SSAGA) [34]. Additional criteria were used to exclude HCP participants from the current analyses, including testing positive for illicit drug use, having a breath alcohol level above zero, use of any tobacco products, irregular menstrual cycles, or head motion > 2.00 mm during the tasks. To attain two groups with equal numbers of men and women, the remaining participants were matched based on age and education level. The final group of participants included 190 individuals (n = 95 per sex, age range 22–36). See Table 1 for detailed participant demographics.

**Table 1:** Participant demographics.

	All	Males	Females
	*n* = 190	*n* = 95	*n* = 95
Race % (n)			
White	69 (131)	67 (64)	71 (67
Black	16 (31)	16 (15)	17 (16)
Other	15 (28)	17 (16)	13 (12)
Age (years)	29.6 ± 0.3	29.6 ± 0.4	29.6 ± 0.4
Education (years)	15.3 ± 0.1	15.3 ± 0.2	15.3 ± 0.2

Data depicts average ± SEM. No differences were found between males and females using t-tests with non-parametric permutation testing via Permutation Analysis of Linear Models (PALM).

To assess for potential menstrual cycle phase effects, we also categorized women to be in either the follicular phase (n = 52) or luteal phase (n = 43) of their menstrual cycle. The regularly cycling women included in this study were considered to be in the follicular phase of their menstrual cycle if the number of days since their last period was 1–12 (for reported menstrual cycle lengths <25 days), 1–14 (for reported menstrual cycle lengths 25–35 days), and 1–17 (for reported menstrual cycle lengths > 35 days). All others were considered to be in the luteal phase of their menstrual cycle.

### Experimental task design

#### Incentive processing

The incentive processing task conducted by the HCP was adapted from Delgado et al. [35] and is described in detail in Barch et al. [36]. Participants were shown a card with a question mark (“?”) and were told the number on the back of the card was between 1–9. Participants guessed whether the number was either lower or higher than 5 by pressing one of two buttons on a response box. Participants were then given feedback to indicate whether they were correct and won money or incorrect and lost money. Although participants believed that they were correct or incorrect based on the accuracy of their responses, feedback was predetermined to create either a reward trial, a punishment trial, or a neutral trial. Feedback was either a green upward facing arrow and “+$1.00” to indicate a reward, a red downward facing arrow and “-$0.50” to indicate a punishment, or a bidirectional gray arrow pointing left and right and the number “5” to indicate neither a reward nor punishment (neutral trial). The experimental design was a block design with two 3 min 12 s runs. Each run had four 28 s blocks (2 reward blocks and 2 punishment blocks) interleaved with a 15 s fixation cross. Each block consisted of eight 3.5 s trials with presentation of the “?” for 1.5 s, feedback for 1.0 s, and an inter trial interval (ITI; fixation cross) for 1.0 s. Reward blocks consisted of 6 reward trials pseudo randomly interleaved with 2 out-of-set trials (1 neutral and 1 punishment trial, 2 neutral trials, or 2 punishment trials). Punishment blocks consisted of 6 punishment trials pseudo randomly interleaved with 2 out-of-set trials (1 reward and 1 neutral trial, 2 neutral trials, or 2 reward trials). Upon completion of the task, all participants were given a standardized amount of money. Since reward and punishment conditions were predetermined and not based on the accuracy of participant responses, there is no behavioral accuracy to be reported.

#### Working memory

The working memory task conducted by the HCP is described in detail in Barch et al. [36]. Participants were shown pictures as part of a 0-back working memory task and a 2-back working memory task. For the 0-back working memory task, a target cue was presented at the start of each block and participants were instructed to press a button on a response box whenever the stimulus was the same as the target cue. For the 2-back working memory task, participants were instructed to press a button on a response box whenever the current stimulus was the same as the stimulus two back. Stimuli were pictures of faces, places, tools and body parts, and each stimulus type was presented in separate blocks. The experimental design was a block design with two 5 min 1 s runs. Each run had eight 25 s blocks interleaved with a 15 s fixation cross. Within each run, ½ of the blocks used the 0-back working memory task and ½ of the blocks used the 2-back working memory task. Each block consisted of ten 2.5 s trials with presentation of the picture stimulus for 2 s, followed by a fixation cross ITI for 500 ms. At the start of each block, a 2.5 s cue indicated the task type (and target stimulus for 0-back).

### fMRI data acquisition

HCP data were acquired with a 32-channel head coil on a Siemens 3T Skyra modified to include a Siemens SC72 gradient coil to achieve a maximum gradient strength of 100 mT/m. Whole brain images were acquired using echo-planar imaging (EPI) with the following parameters: repetition time (TR) = 720 ms, echo time (TE) = 33.1 ms, flip angle = 52°, BW = 2290 Hz/Px, in-plan FOV = 208 x 180 mm, 72 slices, 2 mm isotropic voxels, with a multi-band acceleration factor of 8. For each task, there were two runs, one with right-to-left phase encoding and one with left-to-right phase encoding to minimize signal dropout in the combined results. Details of scanner modifications and data acquisition parameters are further described in Ugurbil et al. [37].

### fMRI data preprocessing and analyses

Imaging data were pre-processed and analyzed using FMRIB Software Library (FSL) 5.0.8 (http://fmrib.ox.ac.uk/fsl) [38] and group-level statistical analyses were implemented using Permutation Analysis of Linear Models (PALM) [39]. The current study used the “minimally preprocessed” Quarter 3 release of the HCP data, which included gradient unwarping, motion correction, fieldmap-based EPI distortion correction, brain-boundary-based registration of EPI to structural T1-weighted images, non-linear FNIRT registration to the MNI152, and grand-mean intensity normalization using tools from FSL and Freesurfer [40]. For the current analyses, data was also re-registered to subject specific T1-weighted scans, spatially smoothed with a 4 mm full-width half-maximum Gaussian kernel, and a high-pass temporal filter of 200 s was applied. First-level analyses were conducted on each participant’s individual task runs separately (2 runs per task). Using the general linear model (GLM), task-related regressors (corresponding to either reward or punishment blocks for the incentive processing task or the 0-back and 2-back blocks for the working memory task) were convolved with the gamma hemodynamic response function. For the working memory task, the task-related regressors were modeled to include only the correct trials for each participant. Confound regressors representing motion were also included in the models for each task. Contrasts between each task condition (reward, punishment, 0-back and 2-back) and the baseline fixation were calculated. Lower level individual runs within each task were then combined using a second level fixed effects analysis to generate the average brain activation map for each participant for each contrast. For each contrast of interest, a contrast “subject-series” was then created by concatenating the contrast maps for all subjects together into a 4D (volume x subjects) data matrix for further processing. This was done for each contrast independently.

**Fig 1:**
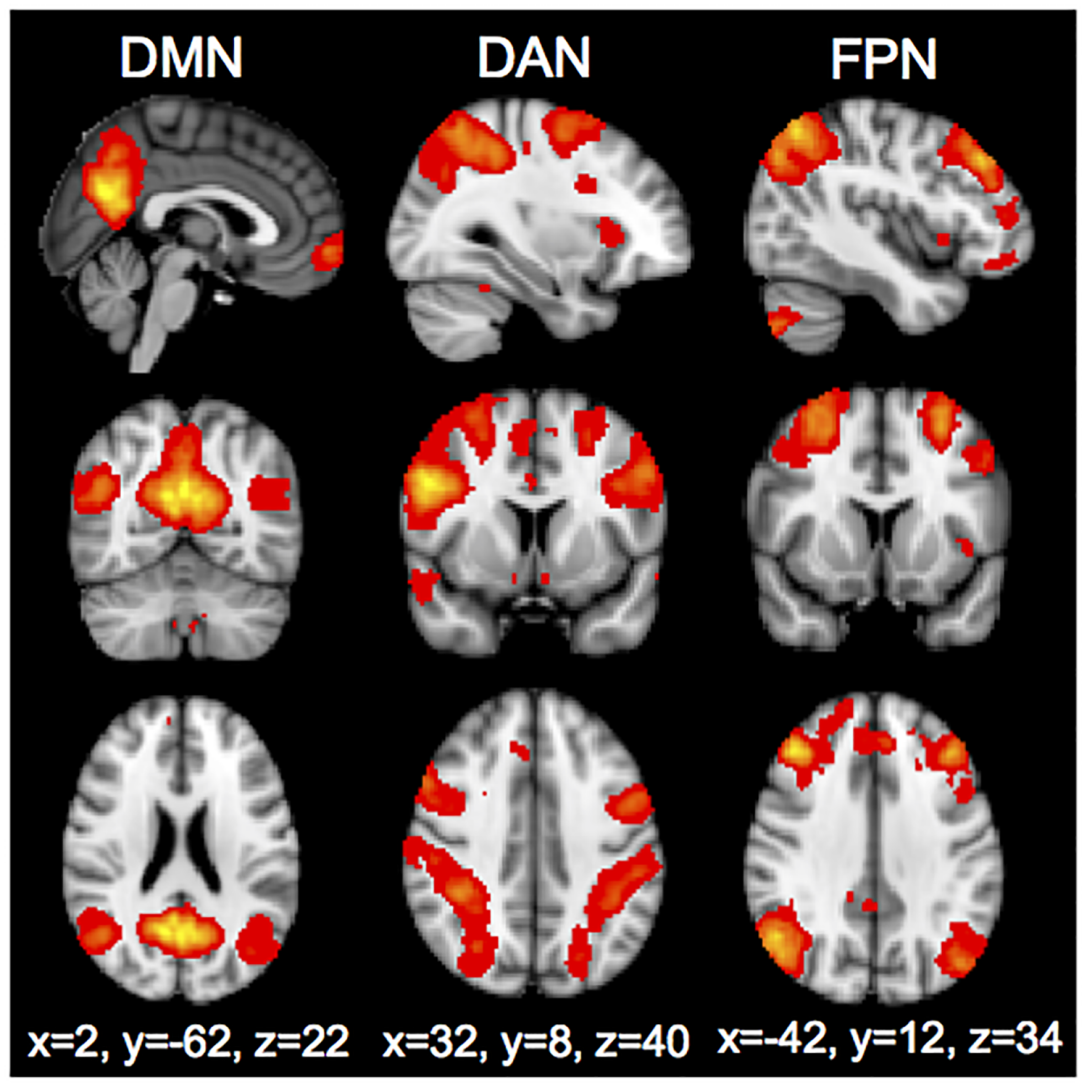
Network ICA maps. Sagittal, coronal and axial images showing the independent components from the MELODIC analysis that correspond to the default mode network (DMN), dorsal attention network (DAN) and frontoparietal control network (FPN). Because two DMN sub-networks and two FPN networks (left and right) were identified, the maps of these networks were added together to show a single DMN and FPN. Networks are shown overlaid on the MNI standard brain image.

To calculate network activation strength, we defined our networks of interest (DMN, DAN, FPN) using independent component analysis (ICA) via FSL’s Multivariate Exploratory Linear Optimized Decomposition into Independent Components (MELODIC) on an independent group of healthy participant resting state scans (see Supporting Information). Brain networks of interest identified from the group ICA are shown in Fig 1. The DMN was comprised of the mPFC, PCC, precuneus, parahippocampus, and lateral parietal lobe. The DAN was comprised of the frontal eye fields, inferior precentral sulcus, dlPFC, middle temporal motion complex, and left superior parietal cortex. The FPN was comprised of the lateral prefrontal cortex, anterior insula, precuneus, middle frontal gyrus, and anterior inferior parietal lobe. Multivariate spatial regression of the template ICA networks against the subject series for each contrast was done to calculate the strength of activation of each network during each task condition (reward, punishment, 0-back and 2-back, each versus baseline fixation) [41]. This analysis results in the network activation strengths for each subject for each contrast.

### Statistical analyses

All statistical analyses were run using t-tests with non-parametric permutation testing via FSL’s PALM to account for family structure in the HCP data. The HCP focused on recruiting twins and siblings, so there is a significant amount of family structure. PALM has specialized functionality for analyzing HCP data, thus this tool was used for inference, and all results are reported with control of family-wise error, p<0.05. Demographic variables (age and education) were assessed using a two-group t-test using PALM (p<0.05, corrected for family structure). Behavioral scores (0-back accuracy and 2-back accuracy) were compared between males and females using a two-group t-test using PALM (p<0.05, corrected for family structure, number of tasks [2: 0-back, 2-back], and number of contrasts [2: males > females, females > males]). For the incentive processing and working memory tasks, sex differences in network activation were assessed for each task contrast using a two-group t-test using PALM (p<0.05, corrected for family structure, number of networks [3: DMN, DAN, FPN], and number of contrasts [2: males > females, females > males]). Influence of menstrual cycle phase (reported at the time of MRI data collection) on network activation was also assessed by a two-group t-test using PALM (p<0.05, corrected for family structure, number of networks [3: DMN, DAN, FPN], and number of contrasts [2: follicular > luteal, luteal > follicular]).

## Results

### Demographic variables and task behavior

As our sample was matched based on age and education, there were no sex differences in either variable (Table 1, p > 0.4 for all contrasts). There were no sex differences in accuracy on the 0-back (males: 91.3 ± 1.1%; females: 91.1 ± 1.0%) or 2-back (males: 84.8 ± 1.0%; females: 81.7 ± 1.2%) working memory tasks (p > 0.05 for all contrasts).

### Sex differences in network activation

#### Incentive processing task

**Fig 2:**
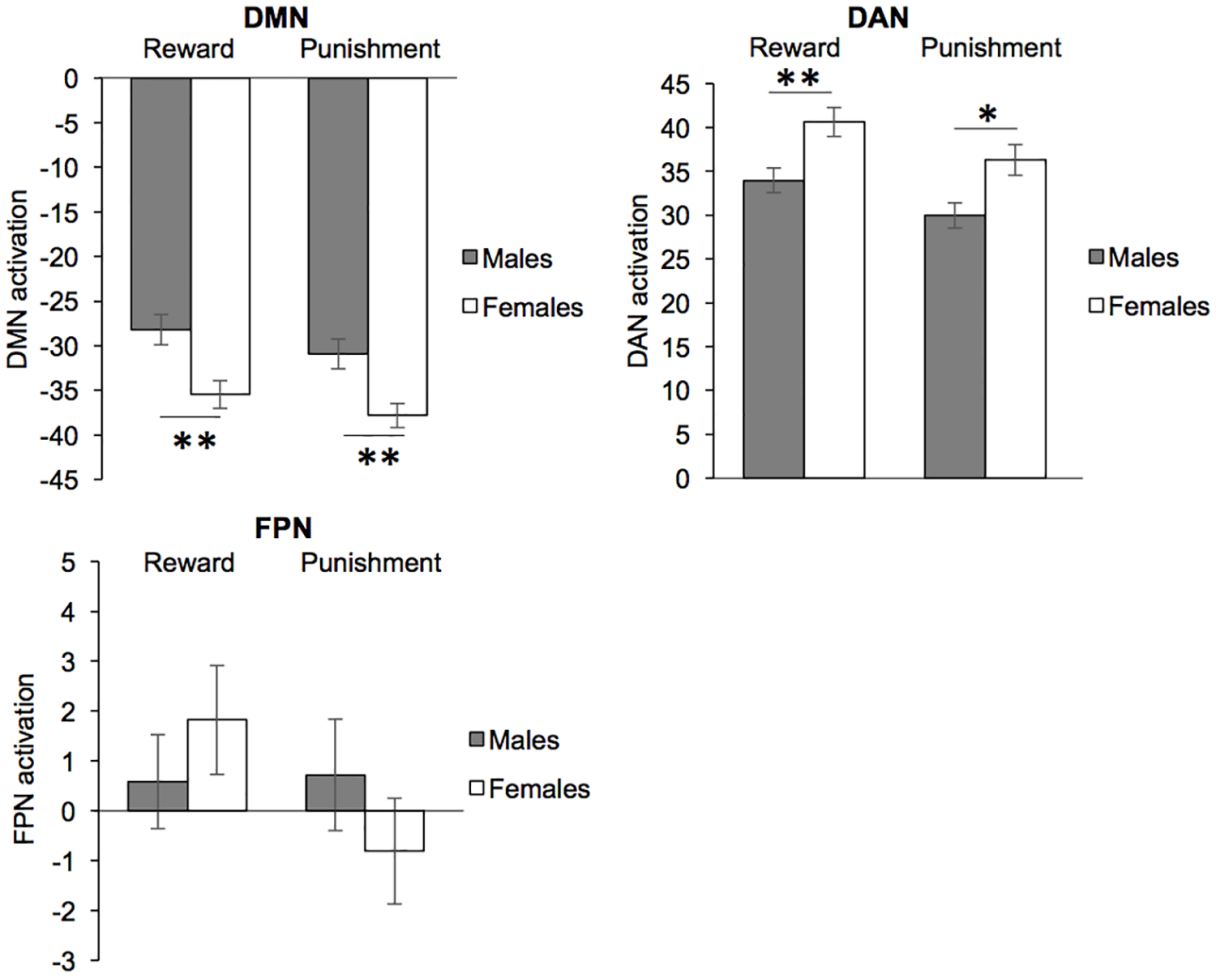
Network activation during reward and punishment trials of the incentive processing task. Females show greater suppression of the default mode network (DMN) and greater activation of the dorsal attention network (DAN) during reward and punishment trials compared to males. There is no sex difference in frontoparietal control network (FPN) activation. * p < 0.05, **p<0.01, t-tests with non-parametric permutation testing via Permutation Analysis of Linear Models (PALM). Bars represent mean ± SEM.

Females showed greater suppression of the DMN during reward (t_(188)_ = 3.16, p = 0.006) and punishment (t_(188)_ = 3.22, p = 0.004) trials compared to males (Fig 2). On the other hand, females showed greater activation of the DAN during reward (t_(188)_ = 3.07, p = 0.008) and punishment (t_(188)_ = 2.80, p = 0.017) trials compared to males (Fig 2). There were no sex differences in FPN activation during either reward or punishment trials.

#### Working memory task

**Fig 3:**
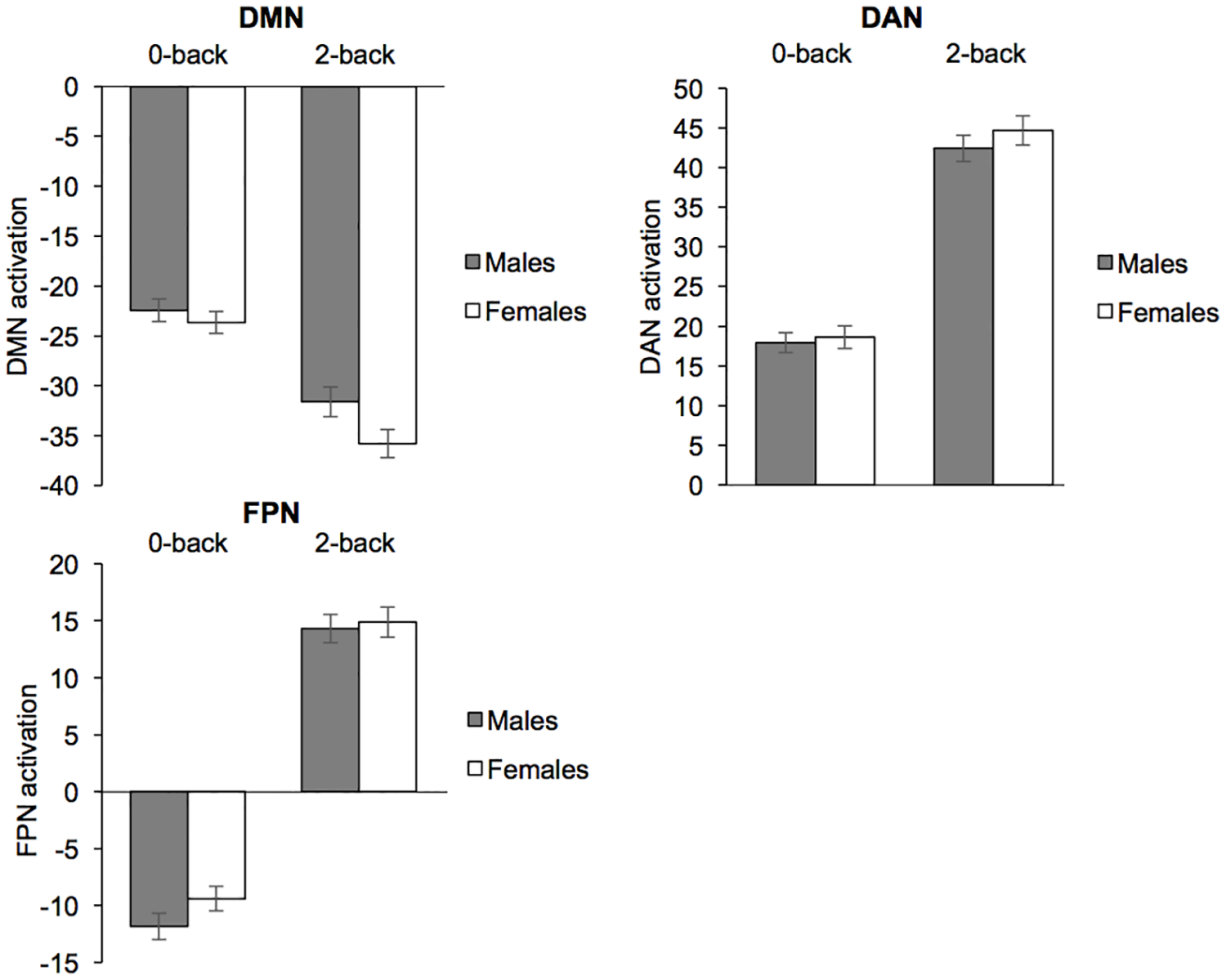
Network activation during the working memory task. There are no sex differences in default mode network (DMN), dorsal attention network (DAN), or frontoparietal control network (FPN) activation during the 0-back or 2-back working memory tasks. Bars represent mean ± SEM.

There were no sex differences in DMN, DAN, or FPN activation during the 0-back (p > 0.3 for all contrasts) or 2-back (p > 0.1 for all contrasts) working memory tasks (Fig 3).

### Menstrual cycle phase effects

When comparing women in the follicular and luteal phases, no differences were found within any of the networks and contrasts of interest for either the incentive processing task (p > 0.5 for all contrasts) or the working memory task (p > 0.3 for all contrasts).

## Discussion

Our results show that women have a pattern of neural activity indicative of enhanced attention to external valenced stimuli. Specifically, during the processing of reward and punishment, but not working memory, women showed increased suppression of the DMN and increased activation of the DAN compared to men. These results suggest that women may have neural processing biases toward stimuli representing reward and punishment.

Greater attention to reward and punishment in women versus men is inferred given the roles of the DMN and DAN in attentional processing. Specifically, the DMN is typically engaged during internally-focused attention, such as self-focused thought, autobiographical memory, and mind wandering [15,42,43]. On the other hand, the DAN plays a role in externally-focused attention, and modulates goal-oriented attentional control [11]. Therefore, greater suppression of internal attentional processes coupled with greater activation of external attentional processes may suggest that women are paying greater attention to reward and punishment stimuli compared to men. These data support previous behavioral and electrophysiological studies that show greater attention to valenced stimuli in women versus men in both healthy [7,10,25,26] and clinical [44] populations. We extend these findings by suggesting that sex-specific attention to valenced stimuli are in part modulated by sex differences in the engagement of the DMN and DAN.

Sex-specific activation of attentional networks during reward and punishment processing may be due to sex differences in reward and punishment sensitivity. Specifically, meta-analyses show that women generally show greater reward and punishment sensitivity compared to men [5,6]. Greater reward sensitivity has been specifically linked with DMN suppression during exposure to reward cues [16]. Therefore, the greater DMN suppression in women in the current study may reflect the heightened reward sensitivity that is found in women versus men.

Though regions of the FPN also play a role in attention [11,45], there was no sex difference in FPN activity during exposure to reward and punishment. A major role of the FPN is to facilitate the interplay between the DMN and DAN. Specifically, the FPN is anatomically interposed between the DMN and DAN [19], and flexibly couples with either the DMN or DAN during internally driven versus externally driven goal-directed cognition, respectively [13]. Therefore, though both the FPN and DAN mediate attentional control, the DAN more directly modulates voluntary goal-directed stimulus response selection and serves to shift and maintain focus on relevant cues [11,27]. Therefore, the greater activation in women versus men in the DAN but not the FPN suggests that women show an enhanced voluntary shift in attention towards reward and punishment. While FPN activation did not differ between the sexes across all tasks, it was suppressed during the 0-back task while activated during the 2-back task and showed relatively little activation during the gambling task. It is plausible that these patterns relate to cognitive load. Specifically, the 2-back task is more challenging than the 0-back task, and FPN regions typically increase in activation during working memory and decision-making tasks [46–48].

The lack of sex differences in network activation during working memory suggests that sex differences in attentional network activity are specific to the processing of valenced stimuli. However, a few studies have found sex differences in DMN activation during cognitive tasks that don’t include valenced stimuli. For example, women showed increased DMN suppression versus men during mental arithmetic [49] and cognitive interference [50]. Because greater DMN suppression is associated with increased task demands and difficulty [18,51], sex differences may emerge when task demands become more challenging for women than for men. Therefore, the mental arithmetic [49] and cognitive interference [50] tasks, but not the working memory task, may have been more challenging for women than men. Indeed, the lack of sex difference in n-back working memory performance in the current study and other studies [28–31] may suggest that the working memory task had a similar level of difficulty for both men and women. However, cognitive effort was not assessed during any of these tasks. Whether sex differences in DMN and/or DAN activation are associated with sex differences in cognitive effort requires further testing.

Knowledge of sex differences in attentional network function during the processing of reward and punishment may help us gain a better understanding of sex biases found in numerous neuropsychiatric disorders. For example, depression and anxiety are more prevalent in women compared to men [52,53]. These disorders are characterized by dysfunctional reward processing [54,55], including attentional bias to negative information [56–58]. It has been hypothesized that women’s enhanced attention to negative information may contribute to their greater prevalence of depression and anxiety [59,60]. It is then possible that sex differences in attentional network engagement during reward and punishment processing may in part underlie this increased vulnerability of women to develop these disorders. Further research is required to test this hypothesis.

There are several limitations of the current work that warrant discussion. First, the HCP dataset was collected to represent a normative sample of relatively young adults and thus there is some heterogeneity in the sample. For example, variables such as a family history of psychiatric/neurological disorders were not considered in the current study. Given the richness of the dataset there are many other variables that could have been taken into account, yet not overly restricting the dataset allows us to suggest the current findings represent sex differences within a general population. Nevertheless, future studies aimed at investigating other factors are warranted. In addition, though we found no difference in network activity between menstrual cycle phases, menstrual cycle was assessed by the HCP via self-report measures, which is an indirect measure of menstrual cycle phase and therefore subject to reporting errors. Given evidence of differences in attentional processing of negative stimuli across menstrual cycle phases [61], further investigation of the effect of menstrual cycle phase on network activation using a more direct assessment of menstrual cycle phase (i.e., hormone measurement) is warranted.

In conclusion, we found that attentional network engagement during the processing of reward and punishment is different in men and women. During exposure to reward and punishment, women showed increased suppression of the DMN, which modulates internally-oriented attention, and increased activation of the DAN, which modulates externally-oriented attention. Therefore, women show a pattern of neural activity that reflects enhanced attention to reward and punishment compared to men. Because of disturbances in reward function that are present in numerous sex-biased psychiatric disorders [54,55,62], a greater understanding of how sex influences reward and punishment processing may give insight into the etiology of these disorders.

## Supporting information

S1 File Supporting information. Details regarding the definition of large-scale networks.
(DOCX)
