# Supplementary material for: Sex differences in default mode and dorsal attention network engagement
Source: PLoS One. 2018 Jun 14;13(6):e0199049. doi: 10.1371/journal.pone.0199049 (PMC6002059; doi:10.1371/journal.pone.0199049)
Supplement: S1 File — Details regarding the definition of large-scale networks. (DOCX) [file pone.0199049.s001.docx]

**Supplement**

**Definition of Large-Scale Networks**

Though the Human Connectome Project (HCP) provides results from applying independent component analysis (ICA) to the HCP resting state fMRI data using FMRIB Software Library (FSL) Multivariate Exploratory Linear Optimized Decomposition into Independent Components (MELODIC), they are provided only at specific dimensionalities that did not capture the individual brain networks as needed for the present study. Because of computational limitations, we were unable to re-run MELODIC at other dimensionalities on the HCP data. For dual regression analyses, a set of template networks does not have to derive from the fMRI data itself, but can be defined by an independent set of subjects (Nickerson et al., 2017). Therefore, in order to control dimensionality to appropriately define our networks of interest, we ran MELODIC on a group of independent healthy participants.

Participants were recruited as part of a study at McLean Hospital’s Imaging Center via online advertisements and assessed by the structural clinical interview for DSM IV-TR. Participants were excluded if they had an organic mental disorder, bipolar disorder, schizophrenia spectrum disorder, current depressive episode, or psychotropic drug use, had breath blood alcohol level greater than zero (Alco-Sensor IV, Intoximeters, St. Louis, MO), or were pregnant. Final participants (n=16, 8 females, average age = 26.75 ± 1.45, age range 19-38) were instructed to remain awake with their eyes open during a 6-min resting state scan. Resting state acquisition was conducted on a Siemens Trio 3T scanner (Erlangen, Germany) using a 32-channel head coil and parameters similar to those used by the HCP: TR = 720 ms, TE = 32 ms, flip angle = 66°, slices 64, voxel size 2.5 isotropic, and a multi-band acceleration factor of 8.

Resting state data were pre-processed and analyzed using FMRIB Software Library (FSL) 5.0.8 (Smith et al., 2004; <http://fmrib.ox.ac.uk/fsl)>. The first five volumes were removed for signal stabilization. Preprocessing included brain extraction, slice time correction, spatial smoothing with a Gaussian kernel for a FWHM of 4 mm, a high-pass temporal filter, and registration to the MNI152 2mm^3^ standard space template (Montreal Neurological Institute, Montreal, QC, Canada). Prior to running the group ICA, each subject’s resting state fMRI data were denoised using a single-subject ICA-based approach. In this case, ICA was done on each subject’s fMRI data with automatic dimensionality estimation, then individual IC maps were visually inspected and those that corresponded to motion and artifacts (assessed by examination of the spatial maps and timeseries; Kelly et al., 2010; Janes et al, 2015, 2018) were regressed out of the pre-processed fMRI data using fsl_regfilt. Once all of the resting state data were denoised, a group-level ICA on the temporally concatenated fMRI data (across all subjects) was run with a dimensionality of 35 to estimate a set of template resting state networks for use in extracting network activation strength from the HCP task fMRI data. Our networks of interest included the DMN, DAN, and FPN. The DMN was split into two DMN sub-networks and the FPN was split into two FPN networks (left and right). Therefore, the average of the activation strengths of the two DMN sub-networks and the average of the two FPN networks were computed to evaluate the activation strengths of a single DMN and FPN. See Figure 1 for images of DMN, DAN, and FPN.
